# Effect of age on the effectiveness of the first-line standard of care treatment in patients with metastatic colorectal cancer: systematic review of observational studies

**DOI:** 10.1007/s00432-019-02948-6

**Published:** 2019-06-14

**Authors:** Mohammed Dagher, Meritxell Sabidó, York Zöllner

**Affiliations:** 1 Hamburg University of Applied Sciences, 21033 Hamburg, Germany; 2 Global Epidemiology Department, Merck KGaA, Frankfurter Str. 250, 64293 Darmstadt, Germany

**Keywords:** Colorectal cancer, Metastatic, Treatment, Systematic review

## Abstract

**Purpose:**

Most metastatic colorectal cancer (mCRC) patients are elderly. This systematic review identifies and describes observational studies evaluating the influence of age on first-line treatment effectiveness in real-world practice.

**Methods:**

Medline and EMBASE were searched up to May 2016. The included studies were those that investigated first-line treatment of mCRC and reported age groups and overall survival (OS), progression-free survival (PFS) or overall response rate (ORR) were included. Studies published before 2008 were excluded. Study quality was assessed using the Newcastle–Ottawa Scale. Data were evaluated by age group (< 70 vs. ≥ 70 years; 65–75 vs. ≥ 75 years) and outcome. A pooled survival median was calculated for patients (cutoff = 70 years).

**Results:**

In total, 11 articles with 11,063 patients were included. Four studies using a cutoff of 70 years of age reported OS and PFS, and two studies reported ORRs. In terms of OS, all studies showed a higher OS for those < 70 years of age than for those ≥ 70 years of age. PFS did not find differences by age. For ORRs, one study favoured the younger group, while the second study did not differ by age. Based on three studies, the pooled medians for  < 70 years of age and ≥ 70 years of age were the same for PFS (10.2) and were 27.0 and 22.9 for OS, respectively. All included studies were of high or acceptable quality.

**Conclusions:**

The results suggest that age has no effect on PFS. For ORR, the results were inconsistent between studies. Younger patients in general had better OS, which might be partly explained by more aggressive treatment. This treatment seemed not to be guided by performance status or number of metastatic sites.

**Electronic supplementary material:**

The online version of this article (10.1007/s00432-019-02948-6) contains supplementary material, which is available to authorized users.

## Introduction

Colorectal cancer (CRC) is the third most common cancer in men (746,000 cases, 10.0% of the total) and the second most common cancer in women (614,000 cases, 9.2% of the total) (Ferlay et al. 2014) worldwide. CRC is the second most common cancer in Europe, accounting for 13.0% of all cancers apart from non-melanoma skin cancers. In 2012, there were 214,866 deaths from CRC in Europe (12.2% of the total number of cancer deaths and the second most common cause of cancer-related deaths) (Ferlay et al. 2015).

The incidence and mortality of CRC increase with age. Approximately, 67% of CRC patients are aged 65 years and older, and the mortality in this age group is 77% (Ferlay et al. 2015). The age of the European population is increasing, with those aged 65 + years estimated to increase from 93.2 million in 2013 to 124.8 million in 2030 (European Commission et al. 2014). Therefore, a considerable increase in the burden of CRC is expected.

Elderly patients (≥ 65 years of age) are underrepresented in clinical trials (Denson and Mahipal 2014; Hutchins et al. 1999; Talarico et al. 2004), including CRC trials (Dotan et al. 2012; Schiphorst et al. 2014). This leads to a lack of evidence regarding the effectiveness of treatment for this population, resulting in heterogeneous recommendations in treatment guidelines for patients with CRC (Dotan et al. 2012; Kaźmierska 2012).

In Europe, approximately 25% of patients with CRC present with the metastatic stage at initial diagnosis, and approximately 50% of CRC patients will develop metastases (Van Cutsem et al. 2014). The ‘most common’ standard of care for first-line therapy consists of adding targeted therapies to chemotherapy agents (with or without tumour resection) (Stein and Bokemeyer 2014; Kelly et al. 2014).

There is a substantial difference in survival across CRC stages (stage I, 97.4% at 5 years; stage IV: 7.5% at 5 years). However, survival estimates present fewer differences between age groups regardless of the stage, including 63.1% at 5 years in the 40–49 years age range, and 58.3% at 5 years in the 70–79 years age range (Cancer Research UK 2015a, b). The elderly might achieve similar survival rates when they are appropriately treated. A meta-analysis of four clinical trials showed that the ≥ 70-year-old group benefits from chemotherapy similar to younger patients (Folprecht et al. 2006). Nevertheless, treatment patterns and guideline recommendations for metastatic CRC (mCRC) vary, especially for the elderly (Dotan et al. 2012; Kelly et al. 2014; Shenoy and Harugeri 2015).

Observational studies might play an important role in providing data regarding the influence of age on the effectiveness of first-line treatment in mCRC in real-world practice (Concato 2012). A systematic review of observational studies was conducted to evaluate whether the effectiveness of the standard of care used as first-line therapy (as targeted therapies and/or chemotherapy regimens) for mCRC differs by age.

## Materials and methods

### Search strategy

The population, exposure, comparison, and outcome (PECO) model was used to develop the inclusion criteria and search terms (Higgins and Green 2011). The population was composed of individuals first diagnosed with mCRC. The exposure was treatment with first-line chemotherapy regimens with targeted therapies. Comparisons were established by age groups. The outcomes were effectiveness outcomes such as overall survival (OS), progression-free survival (PFS), and overall response rate (ORR). The search was performed by an expert in Medline and EMBASE (on STN^®^). All aspects of the search included free text and index term searches. Specific mention of patient age was not included in the search string to avoid losing records. The reference lists of included articles were also manually searched, and authors were contacted when needed. We searched articles and conference abstracts published up to May 2016, regardless of the language of publication or country of study.

### Selection criteria

The identified studies were included if they fulfilled all the following criteria: (1) the study focused on mCRC patients; (2) the patients in the study received first-line treatment defined as chemotherapy (CT) plus targeted therapy (bevacizumab, cetuximab, or panitumumab); (3) the study reported the OS, PFS, or ORR as the study outcome; (4) the study had a cohort or case–control design; (5) the patients in the study were at least 18 years of age; and (6) patient age was reported at least as a categorical variable. Studies published before 2008 were excluded because biomarkers for the drugs of interest were integrated into treatment guidelines only after that year (Bellon et al. 2011; Carter et al. 2015).

### Study selection procedures and data extraction

Duplicates were automatically removed from the search platform (STN^®^). A reviewer (MD) screened the titles and abstracts and classified them as excluded or unsure using a bespoke form. The full texts of those categorized as ‘unsure’ were retrieved, reviewed, and then reclassified as excluded, included, or unsure. At this stage, those studies considered ‘unsure’ were assessed by a second reviewer (MS) who was blind to their status, and discrepancies were resolved through discussion. For data extraction, MD used a standardized data collection form specifically developed for this review that had been previously piloted. Data were extracted in the following categories: (1) study characteristics such as author, year, design, patient follow-up period, and sample size; (2) patient characteristics such as gender, Charlson Comorbidity Index, performance status, primary tumour site, primary tumour resection status, number of metastatic sites, primary metastasis (metachronous vs. synchronous), metastatic resection status and distribution of metastases; and (3) treatment characteristics, including the therapies used and treatment duration.

### Quality assessment

A critical appraisal of the quality of each individual study was performed using the Newcastle–Ottawa Scale (NOS) (Wells et al. 2014), which evaluates the quality based on the following three sections: the selection of the study groups, comparability, and exposure (for case–control studies) or outcome (for cohort studies). Studies were rated using a “star” system with a maximum attainable level of nine stars. Quality was classified as (1) high when the majority of criteria were met, i.e., each section had at least a star and the minimum total stars was eight; (2) acceptable when most of the criteria were met, i.e., only one section without a star, with a minimum of six total stars; and (3) low when either few stars were assigned, or two sections lacked stars.

### Data synthesis and analysis

The age groups identified were reported, although the focus was on the two sets of age groups most frequently used in studies, i.e., < 70 years vs. ≥ 70 years, and 65–75 years vs. ≥ 75 years. Patient and treatment characteristics and effectiveness outcomes in these two age groups were compared.

The definition of the effectiveness outcome provided in each study was maintained. Data synthesis was performed by age group and by outcome of interest (OS, PFS, and ORR). For OS and PFS, the median survival of patients in each age category was pooled using studies that reported the 95% confidence interval (CI) of the outcome of interest (Wei et al. 2015). *P* values were considered statistically significant if they were < 0.05.

## Results

### Article selection

**Fig. 1:**
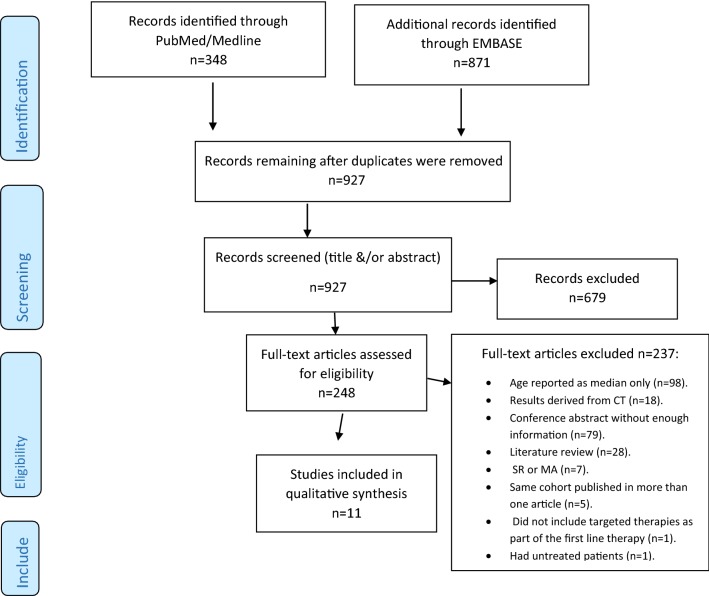
Flowchart depicting the study selection and record screening process

Out of the 1219 articles identified, 971 were excluded because they were duplicates (*N* = 292), out of scope (*N* = 640), or published before 2008 (*N* = 39) (Fig. 1). Therefore, 248 full-text articles were assessed for eligibility. Finally, 11 articles were included, three of which were conference abstracts. The reasons for exclusion were as follows: the patient age was reported chronologically as the median only (*N* = 98), the study results were derived from clinical trials (*N* = 18), the conference abstract had relevant missing information (*N* = 79), the article was a literature review (*N* = 28), the article was a systematic review or meta-analysis (*N* = 7), the same cohort was published in more than one article (*N* = 5), the study did not include targeted therapies as part of the first-line therapy (*N* = 1), and the study had untreated patients (*N* = 1). All included studies were in English, were cohort studies, and had study periods ranging between 2004 and 2015. Supplemental Table 1 describes the study characteristics.

### Patient and treatment characteristics

Overall, the total number of patients was 11,063. Studies included a higher proportion of men than women (Supplemental Table 1). The primary tumour location was mainly in the colon, followed by the rectum. Most patients had one metastatic site, located mainly in the liver. Primary tumour resection before starting chemotherapy was reported in 7 out of 11 studies. The prevalence of prior metastatic resection ranged from 9.1 to 21.0% in three studies (Parakh et al. 2015; Rouyer et al. 2016; Sahm et al. 2016), while the prevalence of metastatic resection after therapy initiation was 17.3% and 22% in Sahm et al. (2016) and Rouyer et al. (2016), respectively. Three studies reported the presence of primary metastases at diagnosis. The proportion of patients with the presence of synchronous metastasis ranged from 58.0 to 81.2%, and it was similar across the different age groups (Kozloff et al. 2010; Slavicek et al. 2014; Parakh et al. 2015).

Supplemental Table 2 displays the median follow-up period, study outcomes, treatment used according to age group, and median duration of therapy. The most frequent patient comorbidities, which were reported by eight studies, were hypertension, cardiovascular diseases, moderate-to-severe renal disease, diabetes, lung diseases, and a history of thromboembolism. The median follow-up period was 22.8 months based on the six studies that reported this information. The median follow-up period was similar in retrospective studies (Dirican et al. 2014; Fourrier-Reglat et al. 2015; Slavicek et al. 2014; Tahover et al. 2015) and prospective studies (Kozloff et al. 2010, 2011; Parakh et al. 2015; Rouyer et al. 2016), with 23.2 months and 22.5 months, respectively. In addition to the targeted therapies, which were bevacizumab or cetuximab, chemotherapy regimens included single-agent therapy (IV 5-fluorouracil or oral capecitabine) and/or combination therapy (mainly FOLFOX, FOLFIRI, XELOX, or XELIRI). Among the 11 included studies, 5 used age categories other than ≥ 70 years vs. < 70 years or 65–75 years vs. ≥ 75 years (Dirican et al. 2014; Fukuchi et al. 2013; Fourrier-Reglat et al. 2015; Parakh et al. 2015; Sahm et al. 2016). The median duration of therapy was reported in seven studies and ranged from 7 (Dirican et al. 2014; Hofheinz et al. 2014) to 9.97 months (Tahover et al. 2015). Five out of these seven studies reported the median duration of therapy by age group. Two studies (Fourrier-Reglat et al. 2015; Slavicek et al. 2014) showed a significantly shorter duration of therapy for elderly patients in comparison with younger patients. The other three studies (Kozloff et al. 2010; Rouyer et al. 2016; Tahover et al. 2015) showed a similar duration of therapy between the different age groups.

### Patients and treatment characteristics according to age

Supplemental Table 3 displays survival outcomes by age in all included studies.

### Patients aged < 70 years vs. patients aged ≥ 70 years

Four studies (Hofheinz et al. 2014; Kozloff et al. 2011; Rouyer et al. 2016; Tahover et al. 2015) stratified their results by age group (< 70 vs. ≥ 70 years) (Table 1). The total population was 3986 (< 70 years: 2871 patients; ≥ 70 years: 1115 patients). Performance status was reported in two studies (Hofheinz et al. 2014; Rouyer et al. 2016), and most of the patients presented a performance status result of 0–1 (< 70 years: 87%; ≥ 70 years: 82%). In both age groups, primary tumour resection was the most frequent intervention (< 70 years: 90%; ≥ 70 years: 88%) and most patients had one metastatic site (< 70 years: 62.7%; ≥ 70 years: 65.1%) (Hofheinz et al. 2014; Rouyer et al. 2016). In Hofheinz et al. (2014) and Tahover et al. (2015), the chemotherapy backbone was reported as mono (5-FU or capecitabine) and doublet therapy (oxaliplatin- or irinotecan-based combination) per age group. Fewer patients ≥ 70 years of age received doublet combinations (76%) than those < 70 years of age (91%).

**Table 1:** Patient and treatment characteristics by age group

Author, year	*N*	Male (%)	Performance status (%)	Primary tumour resection (%)	No. of metastatic sites (%)	Distribution of metastasis (%)	Primary tumour site (%)	Chemotherapy (%)
< 70 vs. ≥ 70 years								
Hofheinz et al. (2014)	1297/480	63/62	0: 39/32 1: 50/52 2 + : 9.5/14.2 Missing: 2/2	93/92	1: 64/67 > 1: 31/26 Missing: 5/7	Liver: 72/69 Lung: 28/28 Bone: 4/2 Others: 27/22	NS/NS	Mono: 8.1/21.7 Doublet: 89.8/76.5
Tahover et al. (2015)	216/92	51.4/50	NS/NS	NS/NS	1: 30.6/29.3	Liver-all: 63.9/67.4 Liver only: 30.6/29.3 Lung-all: 31.9/45.7	Colon: 69.4 /76.1 Rectum: 27.8/22.8 Colon and rectum: 1.4/1.1	Mono: 2.8/25.0 Doublet: 97.1/75.0
Rouyer et al. (2016)	232/119	53/70.6	0–1: 80.2/71.4 2 + : 9.5/15.1 Missing: 10.3/13.4	74.1/69.7	1: 57.8/58.0	Liver: 70.7/75.6 Lung: 34.9/34.5 Peritoneum: 19.8/13.4 Other: 29.7/21.8	Colon: 67.7/73.1 Rectum: 32.3/26.9	FOLFIRI + BV, Continuous treatment: 59.9/50.4 Stop-and-go: 49.6/40.1
Kozloff et al. (2011)	1126/424	42.7/57.3	NS/NS	NS/NS	NS/NS	NS/NS	NS/NS	FOLFOX, FOLFIRI, bevacizumab, CapeOx, XELIRI, 5FU/LV, capecitabine
65–75 vs. ≥ 75 years								
Slavicek et al. (2014)	932/129	63.6/61.2	0: 27.5/31.8 1: 29.9/30.2 2 + : 1.6/4.7 missing: 41/33.3	NS/NS	1: 58.6/62.7 2: 31.8/30.2 > 2: 9.6/7.1	Liver: 63.7/76.7 Lung: 25.8/24.0 Others: 41.8/31.8	Colon: 61.3/ 65.1 Rectum: 38.7/34.9	Mono: 7/31.8 Doublet: 88/63.6 No CT: 1.6/2.3
Kozloff et al. (2010)	533/363	57.4/57.3	0: 38.5/28.7 1: 47.3/48.0 2 + : 7.1/12.6 missing: 7.1/10.7	86.9/83.8	1: 20.6/19.6	Liver: 44.1/45.4 Lung: 19.3/19.3 Others: 15.9/15.7	Colon: 80.9/81.8 Rectum: 18.8/18.2	Mono: 9.5/18.7 Doublet: 79.5/69.1

*N* number of patients

### Patients aged 65–75 years vs. patients aged ≥ 75 years

Two studies (Kozloff et al. 2010; Slavicek et al. 2014) compared the age group of 65–75 years vs. the age group ≥ 75 years (Table 1). The total population was 1957 patients (65–75 years: 1465; ≥ 75 years: 492). Performance status was reported in both studies, although it had a high proportion of missing values, ranging from 7.1% (Kozloff et al. 2010) to 41% (Slavicek et al. 2014). The majority of the patients presented a PS result of 0–1 (65–75 years: 68%; ≥ 75 years: 73%). In Slavicek et al. (2014), a lower proportion of patients in the age group 65–75 years received monotherapy (7%) than that in the age group ≥ 75 years (31.8%). The same pattern was reported in Kozloff et al. (2010), with a lower proportion at 65–75 years of age receiving monotherapy (9.5%) than those ≥ 75 years of age (18.7%). These CT patterns were found regardless of the given targeted therapy.

### Effectiveness of treatment according to age

### Patients aged < 70 years vs. patients aged ≥ 70 years

**Fig. 2:**
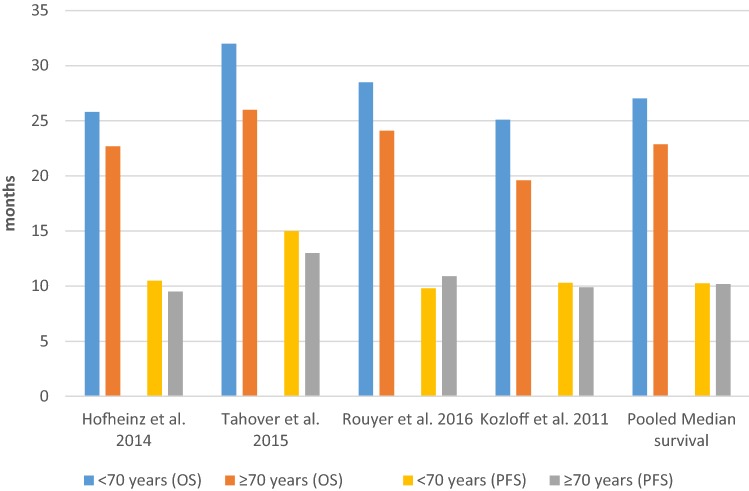
Survival outcomes for age cutoff of 70 years among included studies and estimated pooled median survival. *OS* overall survival, *PFS* progression-free survival

Among the four studies that reported effectiveness results using a cutoff of 70 years of age, all studies reported OS and PFS, and two studies also reported ORR (Table 2). Figure 2 presents the OS and PFS results in patients with a cutoff age of 70 years among the included studies and the estimated pooled median survival.

**Table 2:** Effectiveness of first-line treatment in mCRC patients according to age

	Median follow-up (months)	*N*	Median OS (months) % (95% CI)	Median OS (months) % (95% CI)	*P* value	Median PFS (months) % (95% CI)	Median PFS (months) % (95% CI)	*P* value
< 70 vs. ≥ 70 years		<70/≥ 70 years	< 70 years	≥ 70 years		< 70 years	≥ 70 years	
Hofheinz et al. (2014)	72	1297/480	25.8	22.7	< 0.0008	10.5	9.5	0.074
Tahover et al. (2015)	20.5	216/92	32 (26.1–37.8)	26 (20.8–31.1)	0.093	15 (10.9–19.1)	13 (9.7–16.31)	0.096
Rouyer et al. (2016)	24	232/119	28.5 (25.0–31.0)	24.1 (20.4-26.2)	0.012	9.8 (9.2–11.2)	10.9 (9.4–12.6)	0.51
Kozloff et al. (2011)	21	1126/424	25.1 (23.1–26.9)	19.6 (18.1–21.6)	SG	10.3 (9.8–10.9)	9.9 (8.9–10.4)	NS
65–75 vs. ≥ 75 years		65–75/≥ 75 years	65–75 years	≥ 75 years		65–75 years	≥ 75 years	
Slavicek et al. (2014)	17	932/129	27.5 (25.0–29.9)	25.1 (11.3–38.9)	0.73	11.3 (10.5–12.0)	11.8 (9.6–14.0)	0.94
Kozloff et al. (2010)^a^	20.1	533/363	21.1 (18.6–23.9)	19.2 (16.2–21.1)	NS	9.6 (9.0–10.3)	9.7 (8.5–10.4)	NS

*CI* confidence interval, *N* number of patients, *NS* not significant although not quantified, *SG* significant although not quantified; *OS* overall survival, *PFS* progression-free survival

^a^Kozloff mentioned also the adjusted OS, which was: < 65 years: 24.6 (23.1–26.1); 65–74 years: 22.5 (20.7–24.4); 75–80 years: 20.9 (18.3–23.5); ≥ 80 years: 16.8 (14.8–19.4)

In terms of OS, all studies showed a higher OS among those < 70 years of age than among those ≥ 70 years of age, and three of them reached statistical significance (Kozloff et al. 2011; Hofheinz et al. 2014; Rouyer et al. 2016). Regarding PFS, there was no consistent pattern according to age group, and no study showed a statistically significant difference between the two age groups of interest. For ORR, Hofheinz et al. (2014) showed a significant difference between the two age groups of interest (< 70 years: 62%; ≥ 70 years: 55%, *P* value: 0.0046), while in Rouyer et al. (2016), ORR was similar in both groups (< 70 years: 62.5%; ≥ 70 years: 58.8%, *P* value = 0.947).

Based on three studies that reported the 95% CI of the effectiveness outcomes (Kozloff et al. 2011; Rouyer et al. 2016; Tahover et al. 2015), the pooled median for PFS was 10.25 months for patients < 70 years of age and 10.2 months for those ≥ 70 years of age. The pooled median for OS was 27.04 months for patients < 70 years of age and 22.86 months for those ≥ 70 years of age.

### Patients aged 65–75 years vs. patients aged ≥ 75 years

Two studies reported OS and PFS and not ORR. Both OS and PFS showed similar results between the two age groups of interest (Table 2).

### Quality assessment of included studies

Supplemental Table 3 displays the results of the quality assessment of the studies included using the NOS checklist. The maximum rating per section was four stars in the selection section, two in the comparability section, and three in the outcome section. Two studies (Parakh et al. 2015; Tahover et al. 2015) obtained the maximum possible number of stars (9/9). The study that had the lowest quality obtained six stars (Dirican et al. 2014), and its quality was considered acceptable. Therefore, all the included studies were considered of acceptable or high quality.

## Discussion

This systematic review of the influence of age on survival outcomes in mCRC patients included observational studies that reflect the standard of care in mCRC patient management in real-world practice. A comprehensive search of recently published studies was conducted. All included studies were considered of acceptable or high quality. As a strength, the median duration of follow-up was 22.8 months, which is longer than that reported in clinical trials (18 months) (Lieu et al. 2014).

The focus of the review was on the age cutoff of 70 years, which is the cutoff value most frequently used in studies to define the elderly. In addition, those aged 65–75 years vs. those aged ≥ 75 years were compared. Such a comparison excludes young patients and uses a narrower interval to define the elderly, providing a more accurate picture of the differences among those over 65 years of age receiving the standard of care.

The results suggest that age did not have an effect on PFS and held for all age groups and for the pooled median of PFS using the cutoff value of 70 years. Only one study showed a significantly lower PFS in patients ≥ 75 years of age than in those < 75 years of age (Hofheinz et al. 2014). However, in the same study, PFS did not differ by age when the cutoff value was 70 years. In this study, there appears to be a shift towards monotherapy in the age group from 70 to 75 years, as reflected by the difference in the proportion of monotherapy between the two age groups (≥ 70 years: 21.7%, ≥ 75 years: 31.7%).

OS was the most frequently reported survival outcome (9 out of 11 studies). In most of the studies (*n* = 6, *N* = 6760), the elderly had a lower median OS than the younger age group, regardless of the age cutoff value used (70 years; 65–75 vs. ≥ 75 years). This difference was statistically significant in three studies (Kozloff et al. 2011; Hofheinz et al. 2014; Rouyer et al. 2016). The pooled analysis showed consistent results by age, and the median summary was slightly worse for those ≥ 70 years than for those < 70 years (≥ 70 years: 22.86 months; < 70 years: 27.04 months). These results were consistent with those of the fourth study that we could not combine in our summary analysis (< 70 years: 25.8 months, ≥ 70 years: 22.7 months, *P* < 0.0008) (Hofheinz et al. 2014).

The proportion of patients with synchronous metastasis was similar across the different age groups (Kozloff et al. 2010; Slavicek et al. 2014; Parakh et al. 2015). Nevertheless, one study (Slavicek et al. 2014) showed that the risk of death increased by 25% for synchronous patients in comparison with metachronous patients. In the same study, the risk of disease progression increased by 13% for synchronous metastasis patients. Both results were regardless of age. Furthermore, Parakh et al. (2015) reported that the rate of primary tumour resection (58% vs. 47% vs. 45%, *P* = 0.037) and metastatic resection (26% vs. 21% vs. 6%, *P* < 0.001) for synchronous metastasis patients declined significantly with increasing age (65–74, 75–84, and ≥ 85 years, respectively). This finding might partly explain the significant difference in OS results (65–74 years: 26 months; 75–84 years: 20 months; ≥ 85 years: 11 months, *P* < 0.001) for that study, especially that the median OS of patients who underwent metastatic resection vs. those who did not undergo metastatic resection showed a longer OS for those with metastatic resection in the same age group (65–74 years: 50.4 months vs. 19.8 months, respectively, HR 0.20 (0.13–0.32), *P* < 0.0001; 75–84 years: 37.8 months vs. 20.7 months, respectively, HR 0.26 (0.17–0.39), *P* < 0.001; ≥ 85 years: 20.7 months vs. 10.4 months, respectively, HR 0.43 (0.13–1.36), *P* = 0.15). In the same study, the use of CT declined with increasing age (65–74 years: 84%; 75–84 years: 69%; ≥ 85 years: 34%, *P* < 0.001). Thus, this patient management practice may refer to a treatment practice of using less aggressive treatment with increasing age.

Only two studies showed better OS results in the elderly group than in the younger groups (Dirican et al. 2014; Fukuchi et al. 2013), although these results did not reach statistical significance, and the studies used different age cutoff values (65 and 75 years, respectively). In Fukuchi et al. (2013), patients ≥ 75 years (*n* = 18) had better OS than patients < 75 years (*n* = 108), which might be partly explained by a small number of elderly cases. Both age groups received the same treatment (irinotecan-based CT + bevacizumab). In Dirican et al. (2014), the elderly, defined as patients ≥ 65 years of age, had slightly better OS than those < 65 years of age (31 vs. 22 months, respectively). The design of this study was slightly different from the others; patients (regardless of age) were enrolled in two different cohorts (cohort A: patients were treated with chemotherapy in combination with bevacizumab; and cohort B: patients were treated with the same chemotherapy as cohort A but without bevacizumab). Then, survival results were re-analysed by age group. In both cohorts (with and without bevacizumab), elderly patients showed slightly better results than patients < 65 years of age in terms of OS (< 65 years: 22 months, ≥ 65 years: 31 months) and PFS (< 65 years: 9 months, ≥ 65 years: 11 months). However, in that study, elderly patients represented only 26.8% of cohort A (CT plus bevacizumab), while they represented 52% of cohort B (CT alone). This proportion may reflect the treatment practice for the elderly in comparison with that for younger age groups, especially since all included patients were receiving combination CT (FOLFOX, XELOX, XELIRI, or FOLFIRI regimens). This finding is consistent with the results of other studies (Hofheinz et al. 2014; Kozloff et al. 2010; Parakh et al. 2015; Sahm et al. 2016; Slavicek et al. 2014; Tahover et al. 2015), regardless of the age groups of comparison, in which the intensity of CT and the dose frequency of the targeted therapy decrease with increasing age. Elderly patients tend to receive less combination therapy (FOLFOX and FOLFIRI) and more monotherapy (5-fluorouracil and capecitabine). This treatment pattern was also observed in studies that compared two elderly age groups (65–75 years vs. ≥ 75 years). In those studies, the proportion of patients ≥ 75 years of age receiving monotherapy alone was approximately three times higher than that of patients 65–75 years of age, regardless of the targeted therapy used (Kozloff et al. 2010; Slavicek et al. 2014). Similarly, in another study that reported the use of triple CT backbone by age group (Kozloff et al. 2010), the proportion of patients treated with triple CT backbone dropped progressively with increasing age (< 65 years: 55.3%, 65–75 years: 48.6%, 75–80 years: 32.7%, and ≥ 80 years: 26.1%).

These results might reflect different treatment patterns in the elderly by oncologists, partly explained by a lower tolerance to the adverse events of aggressive CT, which are expected to be more in the elderly than in younger groups. This expectation seems not to be justified by the PS or number of metastatic sites, since the difference observed according to age group was small. The difference in treatment by age might have an influence on the lower OS observed in the elderly. The fact that younger people are expected to live longer might also influence these results.

ORR showed a lower response in the elderly than in younger groups regardless of the age cutoff, although it was statistically significant in only one study (Hofheinz et al. 2014). Fukuchi et al. (2013) showed the opposite results (Fukuchi et al. 2013) with the elderly having a higher response than the younger age group, although this was not statistically significant. This result might be explained by the fact that in Fukuchi et al. (2013), both age groups received the same treatment (irinotecan-based CT + bevacizumab), while in the rest of the studies, treatment varied. The small sample size in Fukuchi et al. (2013), with only 18 patients over 75 years of age, might influence the results reported.

In this systematic review, some limitations should be noted. The search might have missed some studies, although the search strategy was comprehensive and included grey literature. Publication bias might have resulted from the fact that studies with positive results are more likely to be published. The patients’ baseline characteristics showed an imbalance according to age group. The elderly presented a higher frequency of comorbidities and less aggressive treatment, which might have influenced the results. Only 6 out of 11 studies reported the use of propensity score matching to adjust for these baseline differences (Dirican et al. 2014; Fourrier-Reglat et al. 2015; Fukuchi et al. 2013; Hofheinz et al. 2014; Sahm et al. 2016). The included studies did not consider the influence of the histological subtypes on tumour treatment (Hugen et al. 2014). Information on a major potential factor that might influence the outcome for OS was not available or reported in other age categories, as in the case of tumour location, which was analysed in three studies regardless of the age groups (Dirican et al. 2014; Slavicek et al. 2014; Tahover et al. 2015). The study by Slavicek et al. (2014) was the only one to show a significant increasing risk for rectum over colon in both PFS and OS (PFS: rectum/colon, HR 1.10 (1.01–1.19), *P* = 0.04; OS: rectum/colon, HR 1.13 (1.01–1.26), *P* = 0.03). Another factor that impacts OS is the RAS/RAF mutation status. Although the included studies did not refer to the RAS/RAF mutation, we took into account that the biomarkers’ effect on targeted therapies was integrated into treatment guidelines after 2008 (Bellon et al. 2011; Carter et al. 2015). As a result of this, all metastatic patients of the included studies with cetuximab treatment were KRAS wild type (Fourrier-Reglat et al. 2015; Sahm et al. 2016). To reduce heterogeneity, studies with patients receiving only chemotherapy or untreated patients were excluded. Finally, the evaluation of PFS and ORR might differ between oncologists and studies.

## Conclusion

The results of this systematic review suggest that age has no effect on PFS. The ORR suggests slightly better results in younger patients than in the elderly, although the results were highly inconsistent between studies. Younger patients also presented a better OS, which was consistent across most of the age cutoff values. In addition, younger patients received more aggressive treatment than elderly patients. The selection of this treatment pattern seemed to not be guided by the reported PS or the number of metastatic sites given that both parameters differed only slightly by age. Our results suggest that the treatment decision in first-line mCRC should not be guided by the age criterion. Instead, clinical parameters—such as PS, the number of metastatic sites or histological parameters—should play a major role.

Oncologists might face a dilemma. The introduction of more intense therapy in the elderly group might improve their outcomes, mainly OS and ORR. On the other hand, a more aggressive treatment might deteriorate the acceptability of treatment in the elderly by increasing the expected severity of the adverse events, which in turn would worsen their outcomes. Future studies should contribute further evidence to resolving this dilemma by tracking safety across several age groups.

## Electronic supplementary material

Below is the link to the electronic supplementary material.

Supplementary material 1 (DOCX 45 kb)
