# Supplementary material for: Effect of age on the effectiveness of the first-line standard of care treatment in patients with metastatic colorectal cancer: systematic review of observational studies
Source: J Cancer Res Clin Oncol. 2019 Jun 14;145(8):2105–14. doi: 10.1007/s00432-019-02948-6 (PMC6658416; doi:10.1007/s00432-019-02948-6)
Supplement: Supplementary file 1 — Supplementary material 1 (DOCX 45 kb) [file 432_2019_2948_MOESM1_ESM.docx]

**Supplemental Table 1. Characteristics of the included studies**

| **Author, year** | **Cohort design,**  **study period** | **N** | **Male (%)** | **Charlson comorbidity index**  **(%)** | **Performance status (%)** | **Resection of primary tumour/metastasis**  **(%)** | **Number of metastatic sites**  **(%)** | **Primary metastases at diagnosis (%)** | **Distribution of metastasis**  **(%)** | **Primary tumour site**  **(%)** |
| --- | --- | --- | --- | --- | --- | --- | --- | --- | --- | --- |
| Parakh, et al. 2015 | Prospective  07.09-06.14 | 821 | 59 | 0: 49  1-2: 37  ≥3: 14 | 0-1: 74  ≥2: 26 | 52.0/21.0 | NS | Yes: 58  No: 42^[[1]](#footnote-1)^ | Liver: 62  Lung: 32 | Rectum: 24  Colon: 76 |
| Slavicek, et al. 2014 | Retrospective  07.11-09.12 | 3187 | 63 | NS | 0: 28.5  1: 27.2  ≥2: 1.4  Missing: 42.7 | NS/NS | 1:56.7  2: 31.3  >2: 12.0 | Yes: 60.8  No: 39.2^[[2]](#footnote-2)^ | Liver: 64  Lung: 24.3 | Colon: 61  Rectum: 39 |
| Sahm, et al. 2016 | Prospective  06.10-07.15 | 456 | 67 | 0: 54.4  ≥1: 45.6 | 0: 34.4  1: 49.6  2: 8.8  Unknown: 7.2 | NS/15.6 | NS | NS | Liver: 70.6  Lung: 23.5 | Colon: 57.9  Rectum: 42.1 |
| Fourrier-Reglat et al. 2015 | Retrospective  01.09-12.10 | 389 | 67 | NS | 0-1: 77.9  ≥2: 18.0  Missing: 4.1 | 55.9/NS | 1: 53 | NS | Liver: 35.3 | NS |
| Hofheinz et al. 2014 | Prospective  01.05-06.09 | 1777 | 62 | NS | 0: 36.9  1: 50.5  ≥2: 10.8  Missing: 1.8 | 93.0/NS | 1: 64.4  >1: 29.8  Missing: 5.8 | NS | Liver: 71.3  Lung: 27.6 | NS |
| Tahover et al. 2015 | Retrospective  01.05-11.12 | 308 | 51 | NS | NS | NS/NS | 1: 30.2  >1: 70.8^[[3]](#footnote-3)^ | NS | Liver: 64.9  Lung: 36.0  Other: 47.7 | Colon: 71.4  Rectum: 26.3  Colon and rectum: 1  Appendix: 1.3 |
| Rouyer et al. 2016 | Prospective  01.06-12.07 | 351 | 59 | NS | 0-1: 77.2  2+: 11.4  Missing 11.4 | 72.7/9.1 | 1: 57.8  >1: 42.2 | NS | Liver: 72.3  Lung: 34.7  Peritoneum: 17.7  Other: 27.0 | Colon: 69.5  Rectum: 30.5 |
| Kozloff et al. 2011 | Prospective  11.06-01.08 | 1550 | 57 | NS | 0: 49.1  1: 42.5  ≥2: 7  Missing: 1.4 | 80.8/NS | NS | NS | NS | Colon: 76.1  Rectum: 23.6  Missing: 0.3 |
| Kozloff et al. 2010 | Prospective  02.04-06.05 | 1953 | 56 | NS | 0: 50.0  1: 37.7  ≥2: 4.9  Missing: 7.4 | 83.4/NS | NS | Yes: 81.2  No: 18.8^[[4]](#footnote-4)^ | Liver: 58.8  Lung: 18.8  Other: 14.1 | Colon: 77.9  Rectum: 22.1 |
| Dirican et al. 2014 (Gr. A)^[[5]](#footnote-5)^ | Retrospective  01.06-01.12 | 145 | 63 | NS | 0-2: 100.0 | NS/NS | ≤2: 73.8  >2: 26.2 | NS | Liver: 21.4  Lung: 10.3  Lung and liver: 13.1  Other: 17.2 | Rectum: 34.5  Colon: 57.2  Junction: 7.6 |
| Fukuchi et al. 2013 | Retrospective  01.06-12.10 | 126 | 60 | NS | 0: 52.4  1: 42.0  2: 5.6 | 73.8/NS | 1: 50.8  2: 34.1  >2: 15.1 | NS | NS | Colon: 66.7  Rectum: 33.3 |

N: Number of patients; NS: Not specified

**Supplemental Table 2. Study outcomes, age groups, and treatment used by age group**

| **Author, year** | **Outcomes** | **Age groups** | **1st line therapy (regardless of surgeries)** | **Treatment by age group** |
| --- | --- | --- | --- | --- |
| Parakh et al. 2015 | OS | 65 -74, 75 - 84, ≥85 | Oxaliplatin-based, irinotecan-based, single-agent IV 5-fluorouracil, single-agent oral capecitabine, bevacizumab | 65-74: CT comb. alone (31.2%), CT mono alone (7.6%), CT comb+Bev (50%), CT mono+Bev (10.2%), Bev alone (1%). 75-84: CT comb alone (21.3%), CT mono alone (7.6%), CT comb+Bev (33.2%), CT mono+Bev (14.8%), Bev alone (1.2%). ≥85: CT comb alone (19.4%), CT mono alone (52.9%), CT comb+Bev (8.3%), CT mono+Bev (19.4%), Bev. alone (0). |
| Slavicek et al. 2014 | OS, PFS | <65, 65 - 75, ≥75 | FOLFOX, XELOX, FOLFIRI, XELIRI, Capecitabine, 5-FU/LV, Other, Bevacizumab | <65: CT comb+Bev (93.3%), CT mono+Bev (2.8%), Bev alone (0.8%). 65-75: CT Comb+Bev (88%), CT mono+Bev (7%), Bev alone (1.6%). ≥75: CT comb+Bev (63.6%), CT mono+Bev (31.8%), Bev alone (2.3%). |
| Sahm et al. 2016 | PFS, ORR | ≤65, >65-75, ≥75 | Irinotecan-based CT, oxaliplatin-based, 5-FU/FA or capecitabine alone, cetuximab | In total cohort: FOLFIRI (58.6%), FOLFOX (33.6%), mono (7.7%). ≤75: FOLFIRI+Cetux (59.7%), FOLFOX+Cetux (35.4%), Mono+Cetux (4.9%). >75 FOLFIRI+Cetux (51.5%), FOLFOX+Cetux (22.7%), Mono+Cetux (24.2%). |
| Fourrier-Reglat et al. 2015 | PFS, ORR | ≤70 vs. >70 | Irinotecan-based regimen, oxaliplatin-based, cetuximab | ≤70: FOLFIRI+Cetux (54.3%), FOLFOX+Cetux (36.2%), others+Cetux (9.5%). >70: FOLFIRI+Cetux (56.8%), FOLFOX+Cetux (38.5%), Others+Cetux (4.8%). others are not specified. |
| Hofheinz et al. 2014 | OS, PFS | <70 vs. ≥70, <75 vs. ≥75 | Irinotecan-based regimen, oxaliplatin-based, bevacizumab, 5 FU, capecitabine | All + Bev. <70: mono (5FU/Cap.) 8.1%, doublet (Irinotecan & Oxaliplatin-based CT) 89.8%. ≥70: mono 21.7%, doublet 76.5%. <75: mono 9.5%, doublet 90.5%. ≥75: mono 31.7%, doublet 68.3%. |
| Tahover et al. 2015 | OS, PFS | <70 vs. ≥70 | FOLFOX, FOLFIRI, bevacizumab, CapeOx, 5FU/LV, capecitabine | <70: FOLFOX+Bev (43.5%), FOLFIRI+Bev (19.4%), CapeOX+Bev (6%), FOLFOX-FOLFIRI/FOLFIRI-FOLFOX+Bev (28.2%), 5FU/LV+Bev (0.9%), Capecitabine+Bev (1.9%). ≥70: FOLFOX+Bev (32.6%), FOLFIRI+Bev (20.7%), CapeOX+Bev (7.6%), FOLFOX-FOLFIRI/FOLFIRI-FOLFOX+Bev (14.1%), 5FU/LV+Bev (4.3%), Capecitabine+Bev (20.7%). |
| Rouyer, 2016 | OS, PFS | <70 vs. ≥70 | FOLFIRI, bevacizumab | From those who received FOLFIRI+Bev: <70: (66.1%) and ≥70 (33.9%). |
| Kozloff et al. 2011 | OS, PFS | <70 vs. ≥70 | FOLFOX, FOLFIRI, bevacizumab, CapeOx, XELIRI, 5FU/LV, capecitabine | All regimens were CT+Bev but they are not specified per age group. |
| Kozloff et al. 2010 | OS, PFS | <65, 65-74, >75, 75-80, ≥80 | FOLFOX, FOLFIRI, 5FU/LV, XELOX, capecitabine, bevacizumab, EGFR inhibitors | although the authors did not provide the results quantitatively; FOLFOX: in <65: 61.3%, in ≥80: 37.9%. ≥80: bolus 5-FU/LV (21.1%) & infusional 5-FU/LV (8.7%). ≥80: Cap. (6%), <80: 1%. |
| Dirican et al. 2014 | OS, PFS | <65 vs. ≥65 | FOLFOX, XELOX, XELIRI, FOLFIRI, bevacizumab | no more details. |
| Fukuchi et al. 2013 | OS, PFS, ORR | <75 vs. ≥75 | Oxaliplatin-based (mFOLFOX6, CapeOX, combined use of Bev), fluoropyrimidine-based | <75: Oxaliplatin-based (81%), Oxaliplatin-based+Bev (24.6%). ≥75: Oxaliplatin-based (46%), Oxaliplatin-based+Bev (15.4%). |
| CT: Chemotherapy; Mono: Monotherapy; Comb: Combination therapy; 5FU: Fluorouracil; CapeOX or XELOX: Capecitabine and oxaliplatin; XELIRI: Irinotecan and capecitabine; FOLFOX: Leucovorin calcium (folinic acid), fluorouracil, and oxaliplatin; FOLFIRI: Leucovorin calcium (folinic acid), fluorouracil, and irinotecan H=hydrochloride; Bev: Bevacizumab, Cetux: Cetuximab. | | | | |

**Supplemental Table 3. Survival outcomes by age of all included studies**

| **Author, year** | **N** | **Treatment received** | **Age groups (years)** | **N by group** | **OS**  **Median**  **95% CI** | **p-value** | **PFS**  **Median**  **95% CI** | **p-value** | **ORR** | **p-value** |
| --- | --- | --- | --- | --- | --- | --- | --- | --- | --- | --- |
| Parakh et al. 2015 | 821 | CT+/- Bev | 65-74 | 363 (T 304) | 26 |  | … |  | … |  |
|  |  |  | 75-84 | 352 (T 244) | 20 | <0.001 | … | … | … | … |
|  |  |  | ≥85 | 106 (T 36) | 11 |  | … |  | … |  |
| Slavicek et al. 2014 | 3187 | Bev+CT | <65 | 2126 (T 2109) | 26.9 (25.3-28.5) |  | 11.4 (10.9-11.9) |  | … |  |
|  |  |  | 65-75 | 932 (T 917) | 27.5 (25.0-29.9) | 0.73 | 11.3 (10.5-12.0) | 0.94 | … | … |
|  |  |  | ≥75 | 129 (T 126) | 25.1 (11.3-38.9) |  | 11.8 (9.6-14.0) |  | … |  |
| Hofheinz et al. 2014 | 1777 | Bev+CT | <70 | 1297 | 25.8 | <0.0008 | 10.5 | 0.074 | 62 | 0.0046 |
|  |  |  | ≥70 | 480 | 22.7 |  | 9.5 |  | 55 |  |
|  |  |  | <75 | 1571 | 25.8 | <0.0001 | 10.5 | 0.00019 | 61 | 0.0041 |
|  |  |  | 75≥ | 206 | 20.8 |  | 8.9 |  | 51 |  |
| Tahover et al. 2015 | 308 | Bev+/-CT | <70 | 216 | 32 (26.1–37.8) | 0.093 | 15 (10.85–19.14) | 0.096 | … |  |
|  |  |  | ≥70 | 92 | 26 (20.8–31.1) |  | 13 (9.68–16.31) |  | … |  |
| Rouyer et al. 2016 | 351 | Bev+/-CT | <70 | 232 | 28.5 (25.0-31.0) | 0.012 | 9.8 (9.2-11.2) | **0.51** | 62.5 | 0.947 |
|  |  |  | ≥70 | 119 | 24.1 (20.4-26.2) |  | 10.9 (9.4-12.6) |  | 58.8 |  |
| Kozloff et al. 2011 | 1550 | Bev+CT | <70 | 1126 | 25.1 (23.1-26.9) | **NS** | 10.3 (9.8-10.9) | **NS** | **…** |  |
|  |  |  | ≥70 | 424 | 19.6 (18.1-21.6) |  | 9.9 (8.9-10.4) |  | **…** |  |
| Kozloff et al. 2010 | 1953 | Bev+/-CT | <65 | 1057 | 24.6 (23.1–26.1) |  | 9.8 (9.2–10.3) |  | … |  |
|  |  |  | 65-74 | 533 | 22.5 (20.7–24.4) |  | 9.6 (9.0–10.2) |  | … |  |
|  |  |  | ≥75 | 363 | 19.2 (16.2–21.1) | **NS** | 9.7 (8.5–10.4) |  | … |  |
|  |  |  | 75-80 | 202 | 20.9 (18.3–23.5) |  | 75-80: 10 (8.5-10.5) |  | … |  |
|  |  |  | ≥80 | 161 | 16.8 (14.8–19.4) |  | 8.6 (7.5–9.9) |  | … |  |
| Dirican et al. 2014 | 145 | Bev+CT | <65 | 104 | 22 (15.6-28.3) | **NS** | 9 (7.3-10.6) | **NS** | … |  |
|  |  |  | ≥65 | 38 | 31 (19.7-42.2) |  | 11 (8.5-13.4) |  | … |  |
| Fukuchi et al. 2013 | 126 | CT +/- Bev | <75 | 108 | 17.5 | 0.53 | 13 | **0.44** | 36 | 0.947 |
|  |  |  | ≥75 | 18 | 25.4 |  | 18.7 |  | 44 |  |
| Sahm et al. 2016 | 456 | Cetuximab+CT | ≤65 | 235 | NS |  | 12.9 | **NS** | **57.4** |  |
|  |  |  | >65-75 | 155 | NS |  | 10.2 |  | 50.95 | **NS** |
|  |  |  | ≥75 | 66 | NS |  | 8.1 |  | 33.3 |  |
| Fourrier-Reglat et al. 2015 | 389 | Cetux+CT | **≤70** | 273 | NS |  | **9.2** |  | **…** |  |
|  |  |  | **>70** | 116 | NS |  | **9.5** |  | **…** |  |

**Supplemental Table 4. Results of the quality assessment of the studies included using the Newcastle-Ottawa Scale**

| **Author, year** | **Selection (4)** | | | | **Comparability (2)** | | **Outcome (3)** | | |
| --- | --- | --- | --- | --- | --- | --- | --- | --- | --- |
| Slavicek et al. 2014 | * | * | * | * | * | * | * | * | - |
| Fourrier-Reglat et al. 2015 | * | * | * | * | * | - | * | * | - |
| Sahm et al. 2016 | * | * | * | * | * | - | * | * | - |
| Parakh et al. 2015 | * | * | * | * | * | * | * | * | * |
| Hofheinz et al. 2014 | * | * | * | * | * | - | * | * | - |
| Tahover et al. 2014 | * | * | * | * | * | * | * | * | * |
| Rouyer et al. 2016 | * | - | * | * | - | * | * | * | * |
| Kozloff et al. 2011 | * | * | * | * | * | * | * | * | - |
| Kozloff et al. 2010 | * | * | - | * | * | * | * | * | * |
| Dirican et al. 2014 | * | * | * | * | - | * | - | * | - |
| Fukuchi et al. 2013 | * | * | * | * | * | - | * | * | * |

1. Synchronous presentation across age-groups in Parakh et al. (2015) were 58% vs. 53% vs. 57% for 65-74, 75-84, and ≥85 years, respectively. [↑](#footnote-ref-1)
2. Synchronous presentation across age-groups in Slavicek et al 2014) were 62.2% vs. 58.3% vs. 57.4% for <65, 65-75, ≥75 years, respectively. [↑](#footnote-ref-2)
3. Tahover et al. (2015) reported this data as the following: liver all 64.9% (liver only 30.2%, liver and ≥1 other site(s) 34.7%), lung all 36%, others 47.7%. [↑](#footnote-ref-3)
4. Synchronous presentation across age-groups in Kozloff et al. (2010) were 17.7% vs. 20.6% vs. 18.8% vs. 20.5% for <65, 65-<75, 75-<80, ≥80 years, respectively. [↑](#footnote-ref-4)
5. This study consists of two groups (A & B). We are including the information for group (A) only (patients were treated with chemotherapy in combination with bevacizumab). [↑](#footnote-ref-5)
